# Machine learning for the detection of social anxiety disorder using effective connectivity and graph theory measures

**DOI:** 10.3389/fpsyt.2023.1155812

**Published:** 2023-05-09

**Authors:** Abdulhakim Al-Ezzi, Nidal Kamel, Amal A. Al-Shargabi, Fares Al-Shargie, Alaa Al-Shargabi, Norashikin Yahya, Mohammed Isam Al-Hiyali

**Affiliations:** ^1^Centre for Intelligent Signal & Imaging Research (CISIR), Electrical and Electronic Engineering Department, Universiti Teknologi PETRONAS, Bandar Seri Iskandar, Perak, Malaysia; ^2^College of Engineering and Computer Science, VinUniversity, Hanoi, Vietnam; ^3^Department of Information Technology, College of Computer, Qassim University, Buraydah, Saudi Arabia; ^4^Faculty of Engineering, Abu Dhabi University, Abu Dhabi, United Arab Emirates; ^5^Department of Information Technology, Universiti Teknlogi Malaysia, Skudai, Malaysia

**Keywords:** EEG, graph theory analysis, social anxiety disorders, machine learning, effective connectivity, partial directed coherence, support vector machine, event related potential

## Abstract

**Introduction:**

The early diagnosis and classification of social anxiety disorder (SAD) are crucial clinical support tasks for medical practitioners in designing patient treatment programs to better supervise the progression and development of SAD. This paper proposes an effective method to classify the severity of SAD into different grading (severe, moderate, mild, and control) by using the patterns of brain information flow with their corresponding graphical networks.

**Methods:**

We quantified the directed information flow using partial directed coherence (PDC) and the topological networks by graph theory measures at four frequency bands (delta, theta, alpha, and beta). The PDC assesses the causal interactions between neuronal units of the brain network. Besides, the graph theory of the complex network identifies the topological structure of the network. Resting-state electroencephalogram (EEG) data were recorded for 66 patients with different severities of SAD (22 severe, 22 moderate, and 22 mild) and 22 demographically matched healthy controls (HC).

**Results:**

PDC results have found significant differences between SAD groups and HCs in theta and alpha frequency bands (*p* < 0.05). Severe and moderate SAD groups have shown greater enhanced information flow than mild and HC groups in all frequency bands. Furthermore, the PDC and graph theory features have been used to discriminate three classes of SAD from HCs using several machine learning classifiers. In comparison to the features obtained by PDC, graph theory network features combined with PDC have achieved maximum classification performance with accuracy (92.78%), sensitivity (95.25%), and specificity (94.12%) using Support Vector Machine (SVM).

**Discussion:**

Based on the results, it can be concluded that the combination of graph theory features and PDC values may be considered an effective tool for SAD identification. Our outcomes may provide new insights into developing biomarkers for SAD diagnosis based on topological brain networks and machine learning algorithms.

## 1. Introduction

Social anxiety disorder (SAD) is one of the most common mental illnesses recognized by persistent trepidation in different domains of social life (1, 2). Patients with SAD experience deterioration in diverse aspects, including educational life, occupation, and social interactions. They have reported poor quality of life and experience often comorbid psychiatric disorders, such as depression, avoidant personality disorder, and substance abuse (3). The consequences of being affected by SAD are not limited to the patients and their social environment, but they impact the entire social structure, specially through economic costs. A sufficient evaluation of these costs is complicated and, due to the incomplete data, arduous to undertake. Thus, diagnostic prediction for SAD in early time by EEG is a low-cost method and important to prevent complications and rapid worsening.

Recently, shreds of evidence from neuroimaging research have concentrated on characterizing brain circuits of SAD, assisting in a thorough perception of its anatomical and functional substratum (4). These results identified the subcortical hyperactivity impairments associated with SAD neural circuits (e.g., amygdala, insula) and abnormal activities in circuits that control emotional response [e.g., posterior cingulate cortex (PCC), medial prefrontal cortex (mPFC)] (5). The cortical/ subcortical aberration in the neural circuit of SAD is representing the dynamical correlation of emotional pathophysiology and endure threats-related processing bias (6, 7). The neurobiology of SAD indicates that resting-state network (RSN) EEG activity is correlated with many cognitive functions, which allow the investigation of substantial networks (8, 9). Several RSN studies have found that SAD patients show aberrant connections between spatially distant networks indicating baseline disturbances in scattered neural systems. Consistently, SAD patients were found to exhibit an altered RSN of functional connectivity in brain areas including the default mode network (DMN) (10), and the salience network (11). RSN analysis in fMRI permits the detection of brain activity and brain connectomes in the absence of external triggers (12). Nevertheless, findings of brain organization associated with intrinsic spontaneous fluctuations have been found to be limited in fMRI due to its poor temporal resolution and high rate of noise. Therefore, compared to the other existing neuroimaging methods, EEG still remains the cheapest and most prevalent approach to detect brain regions. In particular, its high resolution assists in the monitoring of instant brain activity of cerebral cortex in time and frequency which allows the reflection of substantial neurocognitive networks on a scale of milliseconds (13). Up until now, there has been limited research of EEG to explore brain connectivity patterns of patients with different level of SAD at RSN. Therefore, EEG is selected and used for assessing brain connectivity to the effect of SAD in this study.

Thus Noteworthy, considerable works on traditional EEG signal analysis were used to examine the brain network organization, such as time-series domain, frequency domain, coherence, phase synchronization (14), and mutual information (15). These approaches defined network measures in the analysis of undirected functional connectivity. Functional connectivity has the capability of identifying the connection between brain regions, such as coherence. However, it falls short in revealing the direction and amount of information flow between these regions. The limitations of traditional methods can be overcome by partial directed coherence (PDC). PDC is a frequency-domain metric that provides insight into the direct information flow between two brain areas, insensitive to noise, and unaffected by the influence of other areas. It uses the Granger causality approach to estimate the directed information flow between two signals, while ignoring volume conduction and noise (16). In bivariate cases, PDC and directed transfer function (DTF) are identical (17) (both are EC measures); nevertheless, in the multivariate situation, PDC detects the direct information flows between connected brain regions independent of the impacts of other regions. PDC allows estimating the information flow to comprehend the substrate of cognitive functions by its fundamental use of a multivariate autoregressive model (MVARM) (18, 19). The PDC approach has the merits of permitting the simultaneous modeling of all EEG electrodes with an MVARM, which provides a more precise estimation of causality than a bivariate approach (only two signals are considered at a time). The PDC has been successfully used in many applications including Brain-Computer Interfaces (BCI) (20) and the recognition of topological properties in semantic vigilance, epilepsy (21), and enhanced mental states (22).

Thus far, there was no resting state EEG-based effective connectivity study that is combined with graph theoretical analysis to study the severity of SAD. EEG mapping analysis has demonstrated a decrease in absolute and relative power for all frequency bands and an increment in intermediate beta power in SAD patients compared to HCs. Weighted Phase Lag Index findings exhibited enhanced oscillatory midline connectivity in the theta rhythm in the generalized SAD patients compared to HC group (23). Aberrant EEG connectivity has been found in different mental health conditions that share the same neurobiological features with SAD (24). For Instance, individuals with depression disorder showed greater coherence at RSN compared to HCs [24]. Results are suggesting hyperarousal as a pathogenetic factor of SAD, which may cause the SAD symptomatology (e.g., aberrant cognition, abnormal emotion regulation, functional impairment, deficient memory) (25).

In our previous study, we applied machine learning techniques to study the EEG complexity (26) and deep learning models with PDC features (27) to classify four different subtypes of SAD. Our study found that the deep learning model outperformed the machine learning models, achieving a classification accuracy of 92.86% using a combination of CNN and LSTM, and the most important features for classification were located in the default mode network (DMN) of the brain. However, despite the promising results, our previous study had some limitations, such as the lack of characterization of the stationary behavior of EEG signals that cannot be explained by complexity and brain connectivity. The main objective of the present study is to assess the organization of effective brain networks implicated in different levels of SAD. We also aimed to present a computerized model for EEG-based diagnosis of SAD using a machine learning approach. This analysis concentrates on the application of graph theory measures on the brain connectivity matrices to study the complex underlying behaviors of the brain in SAD. Graph theory analysis is a powerful mathematical approach applied in brain connectivity to identify the organization of brain network patterns (28). The quantification of graph theory metrics permits the characterization of the steady behavior of EEG signals that cannot be revealed by simple linear techniques. Therefore, in this study we quantified the patterns of brain networks over longer time series data by using several graph theory metrics such as clustering coefficient (CC), nodal strength (NS), and nodal degree (ND), local efficiency (LE), and modularity (29). The combination of PDC with graph-theory measures has various advantages for studying SAD. PDC is a powerful tool for assessing influential functional connectivity in the brain, which allows for identifying the direction and strength of information flow between brain regions. However, PDC alone may not provide a complete understanding of the complex network properties of the brain. By combining PDC with graph-theory measures, such as clustering coefficient and segregation, we can better understand the global network properties of the brain and identify the key regions and pathways that contribute to SAD states (30). Graph-theory measures provide information about the organization of brain networks and can reveal patterns of connectivity that are not apparent with PDC alone. Furthermore, integrating PDC with graph-theory measures can increase the specificity and accuracy of identifying biomarkers to better distinguish between healthy and pathological SAD states.

Several studies have applied EEG data to identify and classify certain levels of social anxiety (e.g., HC and anxiety). There have been several previous studies that have used effective connectivity measures such as PDC, Granger causality, Dynamic Causal Modeling (DCM), and directed transfer function (DTF) for SAD classification (31). For instance, one study (32) used Granger causality to investigate effective connectivity between different brain regions and found altered connectivity patterns in individuals with SAD compared to healthy controls. Another study (33) used Dynamic Causal Modeling (DCM) to explore directed functional connectivity between different brain regions in individuals with SAD and found significant differences in connectivity patterns compared to controls. In addition, findings using Weighted Phase Lag Index (WPLI) and graph theory have reported that individuals with generalized SAD exhibited increased oscillatory midline coherence in the theta frequency band during rest in generalized SAD compared to the HCs (23). Similarly, individuals with high trait anxiety exhibited a decrease in theta connectivity between the right medial prefrontal cortex (mPFC) and the right posterior cingulate/retrosplenial cortex, compared to HCs. Additionally, a decrease in beta connectivity was observed between the right mPFC and the right anterior cingulate cortex (34). In comparison to these studies, our study extends the use of effective connectivity measures by incorporating graph theory and machine learning techniques for SAD classification. We used PDC to calculate directed functional connectivity between brain regions and graph theory measures to extract network features from the connectivity matrix. Our machine learning approach allowed us to accurately classify different levels of SAD severity (4 classes) based on the extracted network features. However, analyzing EEG signals to obtain the most discriminative features to segregate different level of SAD remains a challenging task for SAD recognition systems. Accordingly, the fundamental motivation behind this research is to diagnose the severity of SAD and determine whether a person needs to engage with the health care system. Furthermore, we explored whether significant PDC values correlate with self-report measures in SAD. Hence, our main contributions is to the use of directed brain EEG data and graph theory to segregate and classify the severity of SAD into different classes (severe, moderate, mild, and control).

The rest of this paper is organized as follows. In Section 2, the data preprocessing procedure for raw EEG data is detailed, including the steps for computing the EC matrices. The method for extracting features, applying graph theory measures, and using machine learning classifiers is also presented in this section. The results of the statistical analysis and experiments are introduced in Section 3. Moreover, the limitations of the study and a discussion of the results are reported in Section 4. The paper concludes in Section 5.

## 2. Materials and methods

### 2.1. Participants and psychiatric assessment

Eighty-eight participants were selected from a group of 502 respondents who completed the Social Interaction Anxiety Scale (SIAS) self-assessment. These participants were aged between 18 and 25 years, with 36 females (average age 21.97 ± 0.98) and 53 males (average age 22.73 ± 84). None of the participants had a history of psychotropic medication, neurological or surgical disabilities that could affect brain function or metabolism. A sheet containing all study details and a waiver of written informed consent was provided to the selected participants, along with an honorarium for their time and cooperation. This procedure is in accordance with the Helsinki Declaration (35). The study was reviewed, endorsed, and approved by the Medical Science Ethics Committee of the Royal College of Medicine of Perak, Kuala Lumpur University. The Diagnostic and Statistical Manual of Mental Disorders (DSM) was administered by a specialized psychiatrist to provide additional support for the accuracy and reliability of the diagnostic process. The specialized psychiatrist has a comprehensive description of the participants, including their demographic characteristics, clinical status, and relevant comorbidities or medications. The participants' diagnosis was based on the Structured Clinical Interview for DSM-IV (36) and the administered SIAS (37) to determine the severity of SAD. To validate the participants' social anxiety levels during the experiment, we administered the SIAS twice—first during the screening and again after the experiment. We excluded participants who showed a significant difference in SIAS scores between the screening and testing stages. The correlation between the SIAS scores at screening and during testing was found to be high (= 0.87, *p* < 0.0001). In addition, participants were then divided into four categories: control (SIAS score < 20), mild (SIAS score < 35), moderate (SIAS score < 50), and severe (SIAS score < 50). Table 1 summarizes the demographic data and characteristics of the participants.

**Table 1:** Comparison of demographic characteristics in four SAD groups.

**Group**	**Number of participants**		**Total**	**Age (Mean** ±**Std)**		**SIAS score (Mean** ±**Std)**	
	**Female**	**Male**		**Female**	**Male**	**Female**	**Male**
Severe	12	10	22	22.13 ± 2.78	23.11 ± 1.02	67.53 ± 6.21	66.81 ± 5.32
Moderate	7	15	22	21.98 ± 3.11	22.21 ± 1.25	55.7 3 ± 7.81	54.41 ± 6.61
Mild	12	10	22	22.61 ± 2.32	21.71 ± 2.31	38.32 ± 512	37.71 ± 5.81
Control	8	14	22	21.76 ± 1.73	23.62 ± 1.65	14.71 ± 6.74	16.61 ± 7.34

### 2.2. Experimental design

The scalp EEG recordings were conducted completely at the EEG laboratory. Participants were instructed to be seated comfortably, let their minds wander freely with their eyes closed, in a quiet, mid-dark room; EEG data were recorded for approximately 4–6 min and the participants reported no drowsiness or fatigue during the data acquisition. All subjects were not given any instructions but asked to stay fully relaxed. The same experimental paradigm has been performed in this work (38). Real-time EEG data were consistently recorded in RSN using an eegosports amplifier and referential 32-channel shielded cap (ANT Neuro, Enschede, Netherlands) (39).

### 2.3. EEG acquisition and EEG data processing

The EEG data was recorded at a sampling rate of 2048 Hz and was later downsampled to 256 Hz in order to reduce the complexity of data processing and storage. In accordance with the international 10-20 system, thirty electrodes (FP1, FP2, FPz, F7, F8, F3, F4, FC5, FC1, FC2, FC6, Fz, Cz, T8, P7, P8, C3, C4, C3, CP2, CP4, CP1, CP6, CP5, P3, P4, Pz, O1, O2, and Poz) were located on the cerebral cortex with a consistent spatial arrangement and referenced to CPz, with AFz serving as the ground. The raw EEG data were then processed to remove unnecessary and noisy segments. Artifacts caused by eye movements, breathing, power interference, and cardiac movements were visually inspected and removed or corrected using spatial filters. To enhance the signal quality and eliminate high-frequency electrocortical artifacts, signal noise, and low-frequency deflections, a band-pass filter was applied to obtain segments with the best signal between 0.4 and 40 Hz. The electrodes were mounted according to the international standard 10–20 system with impedance maintained below 10 KΩ. The artifact selection process was carried out by identifying block epochs containing artifacts to be used to characterize the artifact patterns. This was followed by a search for other artifacts and the average of all detected artifacts, such as eye blinks, was calculated and labeled as artifact segments. EEG data with a voltage amplitude exceeding ± 100μ*V* was visually removed. The final corrected EEG data were then exported for power spectral analysis PDC analysis using custom scripts and open-source toolboxes in MATLAB (The MathWorks, Inc.). The open-source toolboxes used included EEGLAB (40) for plotting topographical maps and Brain Connectivity Toolbox (BCT) (41) for graph theoretical analysis. In our analysis, we have found that the application of independent component analysis (ICA) is negatively affect the PDC estimation. To compute the directed causal coherence (i.e., PDC) among channel pairs, RSN data was segmented into 3-s epochs (a total of 128 s), which is in the range of other RSN studies (27, 42). We selected the clean epochs (128 s) only that are free of artifacts and other sources of noise, such as eye blinks, muscle activity, and power line noises. By using clean epochs, we made sure that the EEG data is as free of noise and artifacts as possible, and that the neural activity being measured accurately represents the underlying neural processes of interest. The data for each participant was averaged across all segments to obtain the final average values. The average PDC was calculated for each channel, considering the following frequency bands: delta (1–3 Hz), theta (4–8 Hz), alpha (9–12 Hz), and beta (13–30 Hz). We did not include Gamma waves because It has been reported that the brain waves at frequencies (31–256 Hz) are not very sensitive and can provide contradictory results (43).

### 2.4. Partial directed coherence (PDC)

The concept of PDC was proposed by Baccala and Sameshima in 2001 (44), put forward a new frequency-domain method for the description of Granger Causality (GC). The PDC from channel *j* to channel *i* indicates the directional flow of information from one activity site to another. The implementation of PDC algorithm is as follows:


(1) $$\begin{array}{l} Y\left(t\right)={\left[y_{1}\left(t\right),y_{2}\left(t\right),\ldots ,y_{n}\left(t\right)\right]}^{T}, \end{array}$$

is represented by an autoregressive model of order *p* as given in Equation (2)


(2) $$\begin{array}{l} Y\left(t\right)=\overset{p}{\underset{l=1}{\sum }}{\text{A}}_{l}\text{Y}\left(t-l\right)+\varepsilon \left(t\right), \end{array}$$

where,


(3) $$\begin{array}{l} {\text{A}}_{l}=\left[\begin{array}{ccc} a_{11}\left(l\right) & \cdots & a_{1n}\left(l\right)\\ \vdots & \ddots & \vdots \\ a_{n1}\left(l\right) & \cdots & a_{nn}\left(l\right) \end{array}\right], \end{array}$$

is the coefficient matrix at the time lag *l*. *Y*(*t*) represents the weight vector of *m* ROIs of EEG signals at time *t*, matrix *A*(*r*) indicates the *r*^*th*^ order AR parameters, and **ε**(t) represents the measured error that is believed to be an independent Gaussian process with zero mean. When the coefficients of the MVAR model are adequately calculated, *A*(*F*) is determined as follows:


(4) $$\begin{array}{l} A\left(F\right)=\overset{p}{\underset{r=1}{\sum }}{\text{A}}_{r}e^{-i2\pi fr}, \end{array}$$

Therefore, the PDC value from channel *j* to channel *i* can be expressed as follows:


(5) $$\begin{array}{l} PDC_{i_{j}}=\frac{A_{i_{j}}\left(\bar{f}\right)}{\sqrt{{a_{j}}^{-H}\left(f\right){ā}_{j}\left(f\right)}}, \end{array}$$

where, (ā_*i*_)(*f*)(*i*=1, 2, …*M*) represents the *i*^*t*^^*h*^ column of the matrix Ā(*f*) and *PDC*_*i*_*j*__ represents the strength of causal interaction of the information flow from channel *j* to channel *i* at a frequency of *f*. *H*[*f*] is the Hermitian matrix which is equal to $A^{-1}\left[\bar{f}\right]$. Ā_*ij*_(*f*) is the complement of *A*_*ij*_(*f*) and represents the transfer function from *y*_*j*_[*t*] to *y*_*i*_[*t*] being also an element of *A*[*f*] matrix. Finally, *a*_*j*_[*f*] is the *j*^*th*^ column of *A*[*f*] and *PDC*_*i*_ is the *i*^*th*^ row of *PDC*_*ij*_.

The PDC values were computed for each combination and used as an input feature for the classifiers. Features from these frequency bands were chosen to provide optimum accuracy with optimal order. The appropriate order (p) of the model was calculated by the Akaike Information Criterion (AIC) as described in our previous studies (20, 26, 45). In this study, the optimum order is 5. Besides, it has been found that a 5^th^ order model was enough to model appropriately 95% of 2–3 s segments (46).

### 2.5. Graph theory analysis

A graph network (G) was determined as a combination of nodes (i.e., EEG electrodes) that elucidate the associated brain regions by a group of statistical measures of weighted links (i.e., edges). Network analysis is a valuable tool for understanding the structure and function of networks. Global measures provide a single value that summarizes the entire network, while local measures assign a value to each node in the network, generating a vector with the same number of elements as the number of nodes in the network. Prior to network analysis, the weighted directed network matrix must be normalized and should not contain self-connections, i.e., the weight of nodes on the main diagonal should be set to 0. One well-known toolbox for network analysis is the Brain Connectivity Toolbox (BCT), which uses graph theory to measure the properties of networks both globally and locally (41). A number of mathematical measures can be extracted from a graph to characterize the topological architecture of the network. These architectures explain how efficacious information is transferred between the nodes. Graph theoretic analyses used to investigate the brain at rest have showed an important role in illustrating the basis of the brain's genuine and event-related activity. For that, several advantages for neuroscientists to characterize and predict aberration using a network perspective. In our analysis, we derived different metrics from a graph to study the topological characteristics of the network for SAD. These characteristics explain how reliably information is transmitting between different nodes and provide directed and weighted information on the functional segregation and integration of the networks of SAD. The effective brain network properties are computed from the matrices constructed by PDC for each subject. The weight of the links between two different vertices (electrodes) represents the connectivity power of the causal information flow, which assists in distinguishing between vigorous and weak interaction. Weak information flow can be discarded by thresholding as reported in Section 2.6. In this study, the graphical matrices are based on the concepts of Clustering coefficient, Node strength, Node degree, Local efficiency, and modularity and were calculated using open Matlab scripts (41, 47). Here is a brief description about these measures.

#### 2.5.1. Node degree

The degree of a particular node is known as weighted links connected to that node. A node's degree is estimated by segregating it into in-degree (din) to present incoming information flow intensity; and out-degree (dout) to present outgoing information flow intensity; which are expressed as: *Din*=*Aji* and *Dout* = *Aij*, (where *Aij* is not incontrovertibly equal *Aji* and indicates the entry of the adjacency matrices). Here, Nodal degree is calculated as the sum of the in-degree and out-degree of the node. In our analysis, a node with high out-degree measures reflects the cerebral cortex areas that play an essential part in the information transmission and processing. Similarly, a node with high in-degree measures represents the cerebral cortex areas influenced by other cerebral regions. Thus, the overall degree gives an estimate of nodal hubs which can be computed by:


(6) $$D_{tot}=\sum A_{i_{j}}+\sum A_{j_{i}}$$

#### 2.5.2. Node strength

The node strength refers to the sum of the weights of the links that are connected to the node. In networks with directed connectivity, the in-strength is the sum of the incoming link weights, and the out-strength is the sum of the outgoing link weights. The node strengths provide insights into the activation and flow of information in a specific brain region within an efficient brain network. Node strength can be estimated by the following equation:


(7) $$NS_{tot}=\sum A_{ji}+\sum A_{ij}$$

where *A*_*j*_*i*__ is an element weight of the PDC matrix.

#### 2.5.3. Clustering coefficient

The clustering coefficient (CC) estimates network segregation; i.e., the tendency to which a neighboring node's network is clustered together into local specialized regions. We averaged all local CC to produce the graph's clustering coefficient ranging from 0 to 1. The CC computes the profusion of linked triangles in a network. In neuroscience, the CC has been found to be a valuable measure for comprehending function-structure associations in the brain. Mathematically, the graph's CC can be written as:


(8) $$\begin{array}{l} C_{i}=\frac{2n_{i}}{k_{i}\left(k_{i}-1\right)} \end{array}$$

where *k*_*i*_ is the degree of node *i*, and *n*_*i*_ is the number of edges neighbors of *i*.

#### 2.5.4. Local efficiency

Local efficiency (LE) is a segregation estimation of the fault tolerance of a graph network. It emphasizes whether the information transfer between brain regions (nodes) is still coherent when a node is discarded from the network. Higher LE suggests the effectiveness of the network at the regional scales.


(9) $$LE=\frac{1}{\left(N_{G_{I}}(N_{G_{I}-1})\right)}\sum G,K\in N_{G_{I}}$$

where *N* is the number of nodes in graph *G*_*i*_. *G*_*i*_ is the subgraph of graph *G* that includes all neighboring nodes of *i* (excluding *i*).

#### 2.5.5. Modularity

Modularity is one measure of the structure of networks or graphs. It was developed to estimate the intensity of the partition of a graph network into communities. Graph networks with high modularity have robust weights between the brain regions (nodes) within a network but dispersed weights between nodes in different networks. Modularity indicates the density of edges within the network compared with a random distribution of weights between all nodes regardless of the network. The modularity is calculated as:


(10) $$\begin{array}{l} T=\frac{1}{L}\underset{}{\sum }\left[{\text{A}}_{j_{i}}-\frac{k_{i}k_{j}}{l}\right]\delta_{i_{j}} \end{array}$$

where L is the number of edges in the graph, *A*_*j*_*i*__ is the connectivity matrix, *k*_*i*_*k*_*j*_ is the degree of the node *i*.

A complete mathematical demonstration of the graph-theoretical measures can be found in previous works (41, 48, 49).

### 2.6. Creating adjacency matrices

**Figure 1:**
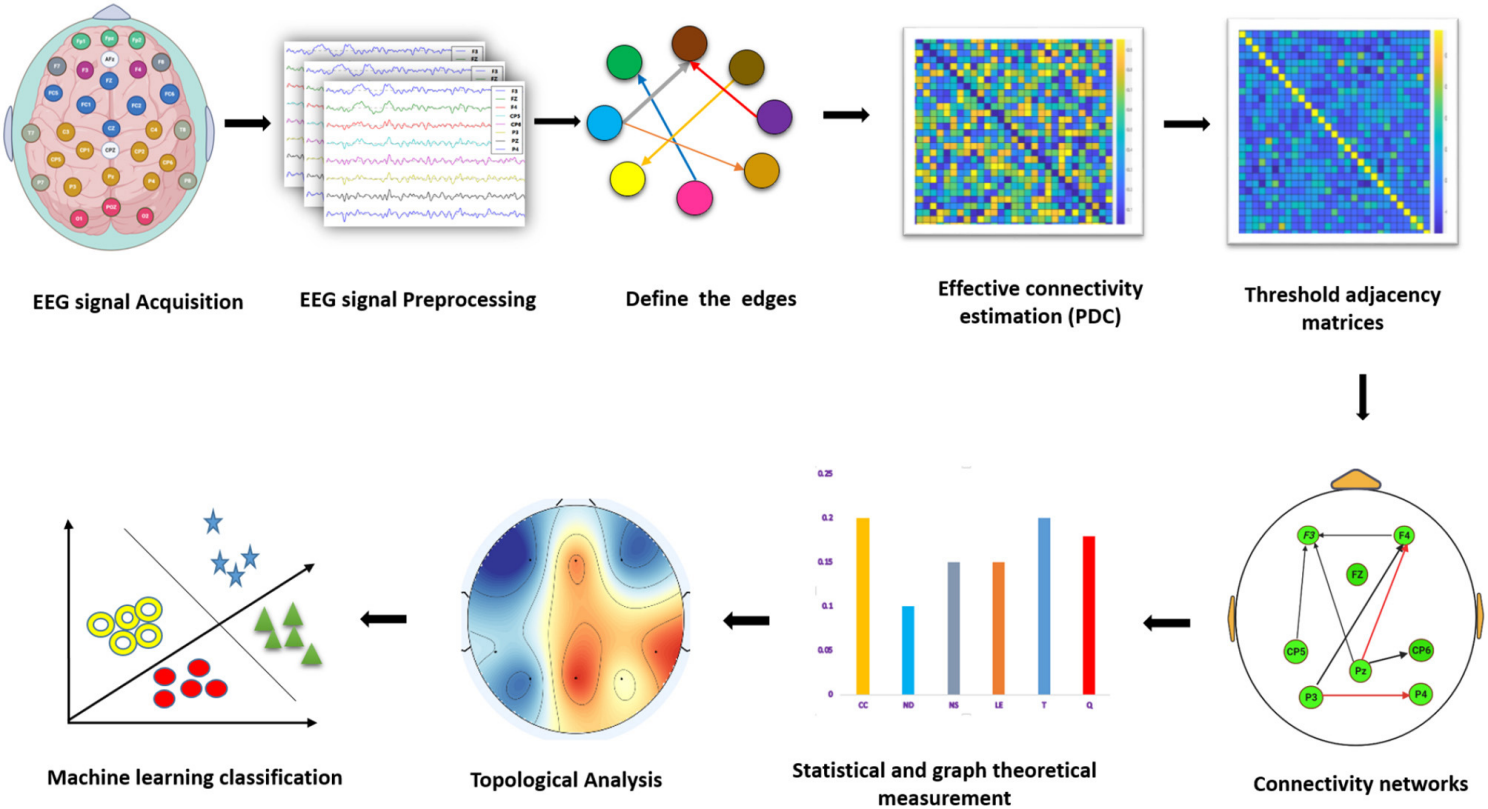
Block diagram for the EEG data analysis module to identify the parameters of the EC network, graph theory measures, and application of machine learning models.

After constructing a network, a conventional implementation is to convert the original weighted network into adjacency matrices, with edges indicating the strength of connections. We performed proportional threshold (PT) on the connectivity matrices of PDC by preserving a proportion *p* (0 < *p* < 1) of the strongest connections (50) and setting these connections to 1, with all other connections set to 0. The selection of the optimum thresholding value was based on global cost efficiency. The absolute value that gives the highest global cost efficiency was selected (51). The steps of data analysis are depicted in the block diagram (Figure 1).

### 2.7. Power analysis and EEG frequency decomposition

The constructed EEG signals and all epoch's parameters are coordinated with PDC and Fast-Fourier transforms. All subject's data were segmented and averaged across all EEG channels and the mean absolute power measures were computed for each of the following frequency bands: Delta (0.5–4 Hz), Theta (4–8 Hz), Alpha (8–12 Hz), Beta (13–30 Hz). A 50% overlapping Hanning window was applied to minimize spectral leakage.

### 2.8. Feature extraction

**Figure 2:**
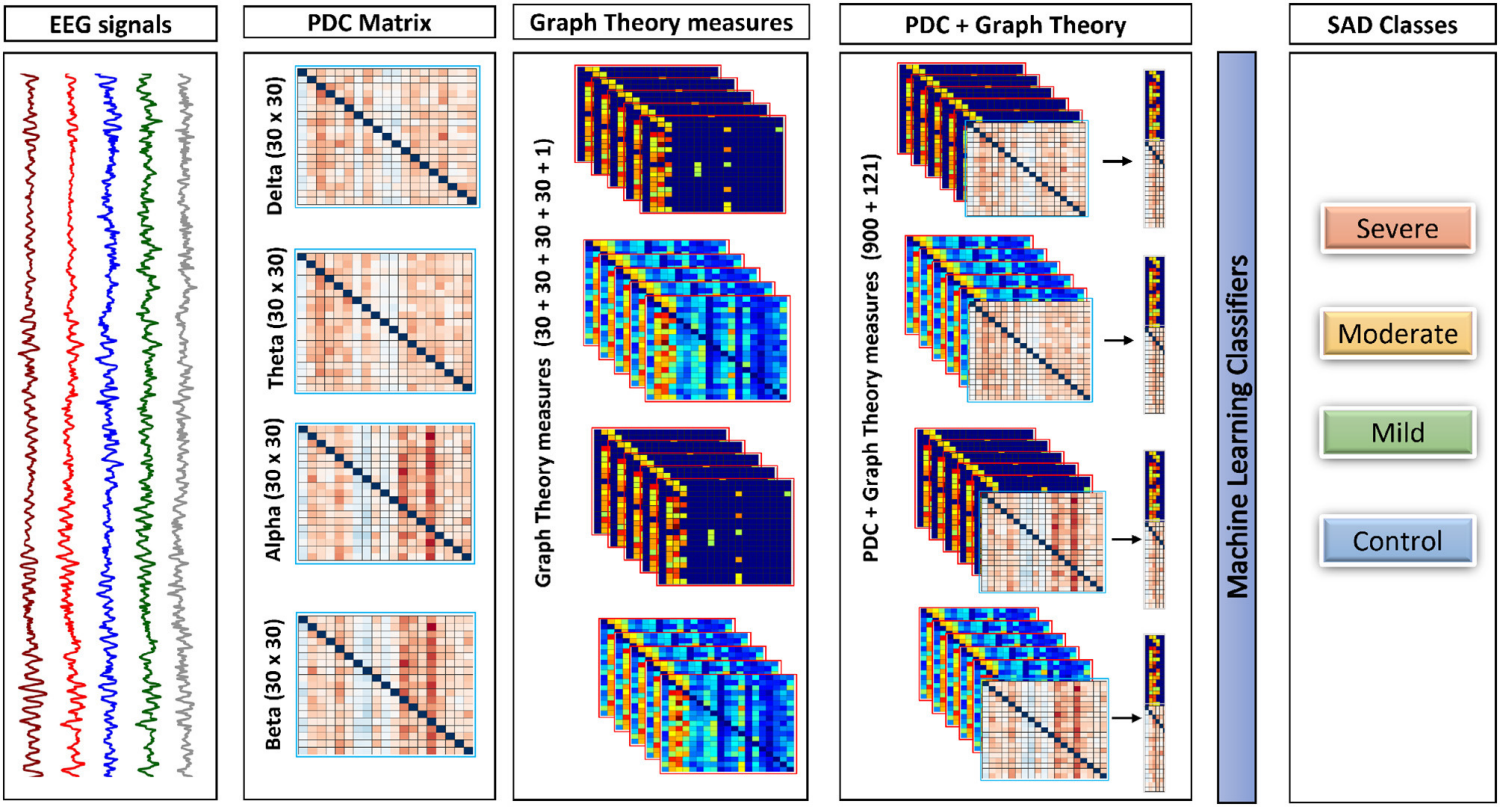
The framework of the proposed approach. This framework shows how process of combining both PDC and graph theory measures to be given as the input to the classifiers. All features in the diagram is representing one subject for one trail (Epoch).

Initially, the PDC values were computed individually in each segment for each subject for every single frequency band, which resulted in a total of 1 × 30 × 30 × 40 (frequency × channels × channels × epochs) weighted and directed connectivity features, which results in a matrix of 4 × 900 × 40. Following that, a set of complex network measures (30 clustering coefficients, 30 local efficiency values, 30 node degrees, 30 node strengths, and 1 modularity), were derived from each PDC network for each frequency band (i.e., 4 frequency bands × 30 clustering coefficients + 30 local efficiency values + 30 node degrees + 30 node strengths + and 1 modularity × 40 epochs), which results in the concatenated matrix of 4 × 121 × 40. We then concatenated the PDC features with the complex network measures in each band and used them as an input to the classifiers with a final matrix of 4 × 1021 × 40. For readability we explain the previous process of concatenating features in Figure 2 to make it more clear and understandable.

### 2.9. Statistical analysis and classification

Classification is believed to be one of the most crucial approaches of supervised learning in which the classifiers learn from the given data to predict new observations. In this study, five machine learning methods are applied; K-Nearest Neighbors (KNN), Linear Discriminant Analysis (LDA), Naïve Bayes (NB), Decision Tree (DT), and Support Vector Machine (SVM). These classifiers were briefly explained previously in (52). All machine learning parameters, such as grid search approach, kernel function, regularization parameter, smoothing parameter, the criterion for splitting, Radial Basis Function (RBF), and the maximum depth of the tree were selected from this work (47). To achieve enhanced performance and an understandable method to compute the prediction quality and constructing an efficient machine learning model, the following three measures are used to examine performance quality:


(11) $$\begin{array}{l} Accuracy=\frac{TP+TN}{TP+FP+TN+FN}\times 100 \end{array}$$

Sensitivity: The sensitivity performance of a test is the possibility of the classifier to differentiate the SAD individual's cases correctly. To compute the sensitivity, we have to quantify the proportion of true positive (TP) in SAD individuals. Sensitivity is calculated mathematically as:


(12) $$\begin{array}{l} Sensitivity=\frac{TP}{TP+FN}\times 100 \end{array}$$

Specificity: The specificity of a test is the possibility of the classifiers to identify the HC subjects correctly. To evaluate the specificity, we have to compute the proportion of true negative (TN) in HC subjects. Specificity is mathematically expressed as follows:


(13) $$\begin{array}{l} Specificity=\frac{TN}{TN+FP}\times 100 \end{array}$$

The application of various machine learning classifiers assists in creating the most appropriate classifier for SAD state diagnosis. In all the applied classification algorithms, we used 10-fold cross-validation to assess the classification accuracy and to reduce the variation of a random segmentation of the dataset. In each classifier, we performed subject-dependent classification with 10-fold cross-validation to estimate the classification accuracy. The chosen features in each subject were randomly and evenly split into 10 equally size subsets. For each of the subsets, we trained the classifiers using nine subsets while the testing was done using the remaining subset. To acquire all predicted labels of all samples, we iterated this procedure 10 times so that each subset is used for validation. Prior to classification, we used Sequential Feature Selection (SFS) algorithm (53) to lower the dimensionality of the EEG features. The SFS generates a set of uncorrelated variables. Thus, it selects a sufficiently reduced subset from the EEG feature space without affecting the performance of the classifier. Cross-validation is a powerful technique for evaluating the performance of EEG classifiers. By repeating this process with different subsets, 10-fold cross-validation can provide an estimate of how well the classifier is likely to perform on new, unseen data. One great advantage of using the 10 cross-validations in our EEG classification is that it helps to avoid overfitting. This provides a more accurate estimate of how well the classifier will perform on EEG data. Another merit of 10-fold cross-validation is that it allows for the optimization of classifier parameters. The mean classification accuracies and standard deviations corresponding to the proposed methods of EEG analysis at the four frequency bands in four classes of SAD are, respectively, computed.

## 3. Result

### 3.1. Participants

Sixty-six individuals diagnosed with SAD and 22 healthy controls participated in the study. The final distribution of the participants was as follows: 22 severe (mean age 22.52 years, Std 2.48), 22 moderate (mean age 23.01 years, Std 1.25), 22 mild (mean age 22.94 years, Std 2.74), and 22 healthy controls (mean age 23.11 years, Std 1.93). Age was not found to significantly differ among the groups, as indicated by a *F*-test [*F*_(1,87)_ = 3.457, *p* = 0.062, $\eta_{{}^{2}}$ = 0.089]. As expected, SAD patients reported higher symptom severity, as measured by the Social Interaction Anxiety Scale (SIAS), compared to healthy controls. The SIAS scores were 67.75 ± 14.34 for severe cases, 48.12 ± 12.51 for moderate cases, 31.82 ± 15.21 for mild cases, and 11.03 ± 9.22 for healthy controls, with all *p*-values less than 0.05.

### 3.2. Power analysis

**Figure 3:**
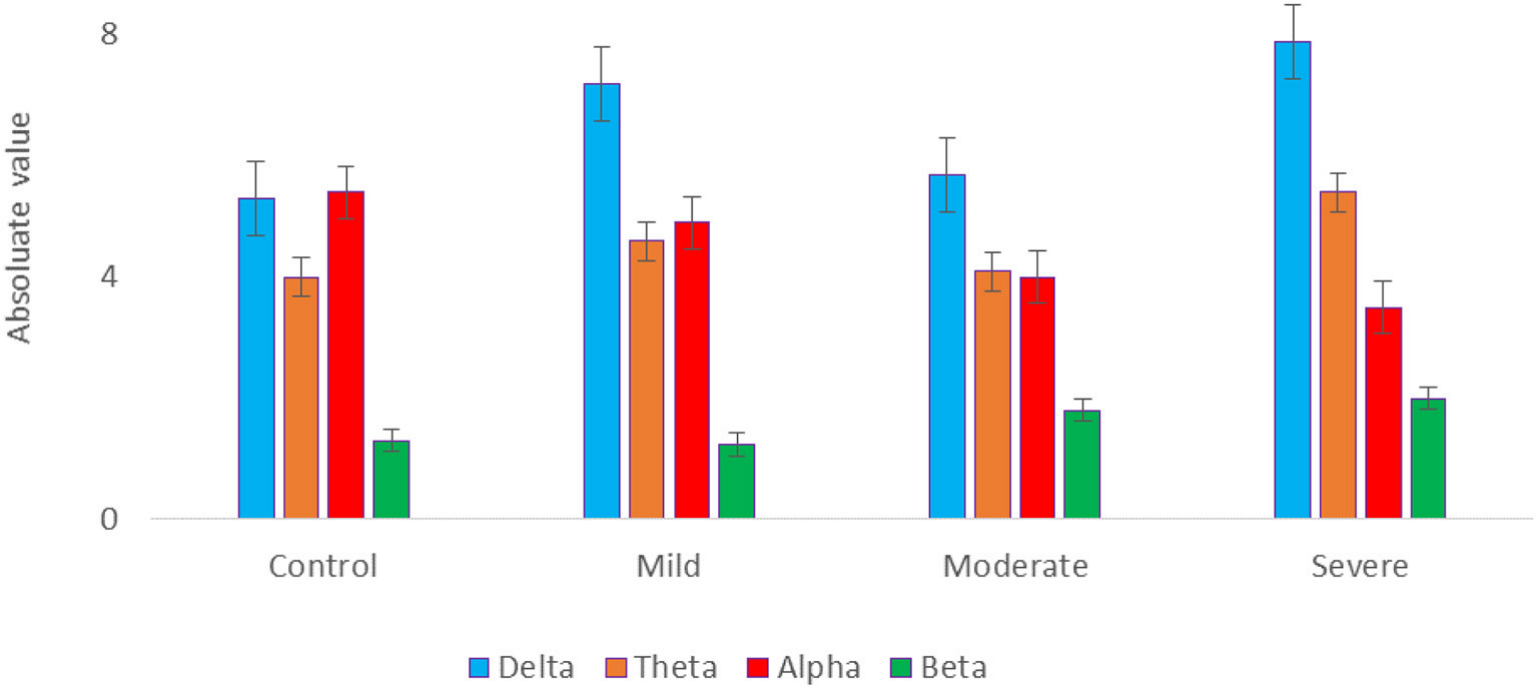
The mean absolute power of all SAD groups in different frequency bands.

The EEG power at resting state for all electrodes was analyzed for 4 groups of SAD patients (severe, moderate, mild, and HC) and 4 frequency bands (delta, theta, alpha, and beta) using a one-way ANOVA with repeated measures on the frequency band factor. There was no significant effect for group or frequency band (all *p* > 0.05) and no group x frequency relationship was found (*p* = 0.134). The ANOVA test revealed a significant impact of emotional state on absolute alpha power, with *F*_(1,119)_ = 3.523, *p* = 0.032, $\eta_{{}^{2}}$ = 0.176. The average absolute power for delta, theta, alpha, and beta in the different SAD severity groups and HC groups can be seen in Figure 3.

### 3.3. Effective connectivity

**Figure 4:**
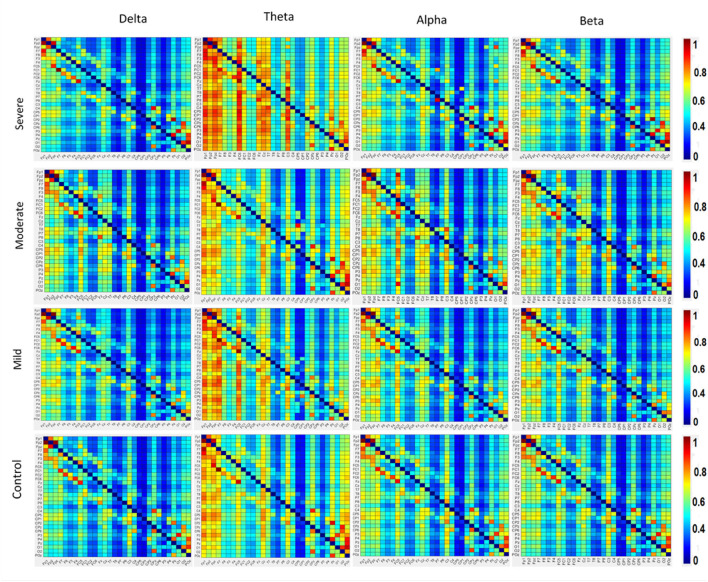
The averaged resting-state PDC intensity of the brain's neuronal clusters. Each element in the matrix represents the mean PDC magnitude between two regions for the SAD groups. The flow of information can be visualized by the direction from the lower rows to the left columns. The red color of the component indicates a greater flow of information between the nodes. The diagonal values are set to zero. All electrodes are in the order of this arrangement: FP1, FP2, FPz, F7, F8, F3, F4, FC5, FC1, FC2, FC6, Fz, Cz, T8, P7, P8, C3, C4, C3, CP2, CP4, CP1, CP6, CP5, P3, P4, Pz, O1, O2, and Poz.

The results in Figure 4 indicate significant differences among the SAD groups in all frequency bands. There was a significant difference in theta, alpha bands, *F*_(3,252)_ = 2.699, *p* < 0.001, $\eta_{{}^{2}}$= 0.105 and, *F*_(3,119)_ = 3.438, *p* < 0.018, $\eta_{{}^{2}}$= 0. 130, respectively. Contrary to the theta and alpha bands, no significant differences were observed in the delta *F*_(3,119)_ = 1.182, *p* < 0.320, $\eta_{{}^{2}}$= 0.030 and beta *F*_(3,119)_ = 2.66, *p* < 0.063, $\eta_{{}^{2}}$= 0.07. The severe SAD group had the highest averaged PDC connectivity across all participants. In theta connectivity, the pre-frontal lobe (e.g., Fp1, Fp2, and Fpz), dorsolateral prefrontal cortex (e.g., F3, F4, and Fz), left frontal cortex (e.g., FC5), midline cortex (e.g., Fz and CZ), posterior cingulate cortex (PCC)/ precuneus (Pz) and left temporal (T7) showed significantly stronger connections to most electrodes in the SAD groups relative to HC group. In addition, severe group showed significant connections in occipital cortex (e.g., Pz, O1, O2, and POz) in alpha band. In particular, these electrodes namely Fp1, Fp2, P3, Pz, P4, P8, O1, and O2 show the highest information flow in RSN (based on the layout of the 31 electrodes in the 10–20 system). Table 2 shows the statistical results for information flow analysis.

**Table 2:** PDC values compassion for different frequency ranges in HCs and SAD groups.

**Frequency**	**Group**	**Mean**	**SD**	***F***	***p*-value**	** $\eta_{{}^{2}}$ **
Delta	Severe	0.121	0.055	1.182	0.320	0.030
	Moderate	0.105	0.041			
	Mild	0.104	0.039			
	Control	0.105	0.016			
Theta	Severe	0.220	0.102	2.699	0.018*	0.105
	Moderate	0.157	0.092			
	Mild	0.172	0.153			
	Control	0.133	0.130			
Alpha	Severe	0.144	0.077	3.488	0.01*	0.130
	Moderate	0.120	0.043			
	Mild	0.109	0.041			
	Control	0.110	0.014			
Beta	Severe	0.135	0.066	2.660	0.051	0.063
	Moderate	0.106	0.041			
	Mild	0.110	0.045			
	Control	0.109	0.0149			

Significant differences were found in theta and alpha bands only (ANOVA test, *p* < 0.05). The asterisk indicates a statistical significance between the means.

Figure 4 also shows the information flow maps of peak activity of EC and the activated areas associated with the three classes of SAD (severe, moderate, mild) and HC in four different frequency bands (delta, theta, alpha, and beta). It can be noted that from Figure 3, with RSN, the connected regions are relatively enhanced in left-brain areas. These findings reveal a similar complex network characterized by numerous connections between frontal, temporal, central, parietal, and occipital regions. The strength of the connection, represented by the weight, determines the amount of information that flows from one node to another.

### 3.4. Graph theory network measures

#### 3.4.1. Node degree

**Figure 5:**
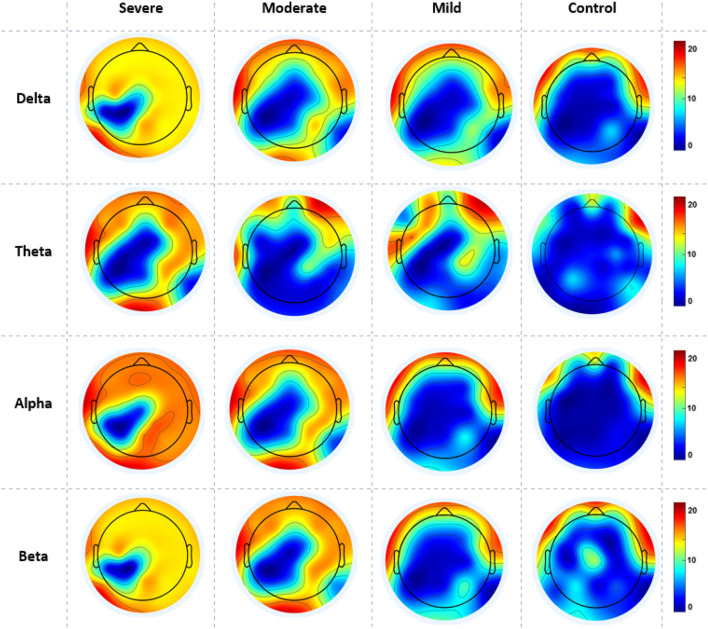
A mean nodal degree in resting-state for all SAD groups (severe, moderate, mild, and HCs) and frequencies (delta, theta, alpha, and beta). Red color indicate higher nodal degree; White color indicate lower nodal degree. The topographic maps were created with EEGLAB function (the venerable topoplot). This includes a 2D grid representation of the scalp and nodal degree values as color-coded dots or contours at the corresponding scalp locations (10-20 system).

The cortical activations displayed on the cortex of averaged ND (in and out degrees) at each electrode during RSN are shown in Figure 5. The ND of connectivity increased in higher SAD severities (severe and moderate groups) in RSN for all frequency bands. However, the ND of directed connectivity in theta and alpha bands have shown significant differences between four SAD groups with (*p* > 0.025) and (*p* > 0.05), respectively. The increase of ND was observed at the medial prefrontal cortex (mPFC), lateral parietal cortex (LPC), PCC, precuneus, ventrolateral frontal cortex (VFC), and occipital cortex. Particularly, in theta and alpha bands, a higher ND of connectivity was observed in the midline frontal cortex and PCC compared to delta and beta. The beta band showed increased ND in the left brain hemisphere [e.g., PFC, LPC, dorsolateral prefrontal cortex (DPC), and PCC]. The moderate SAD group has shown deactivated activities in the LPC and PCC in all frequency bands. Furthermore, mild group showed a decreased ND of connectivity in the mPFC and LPC.

#### 3.4.2. Node strength

**Figure 6:**
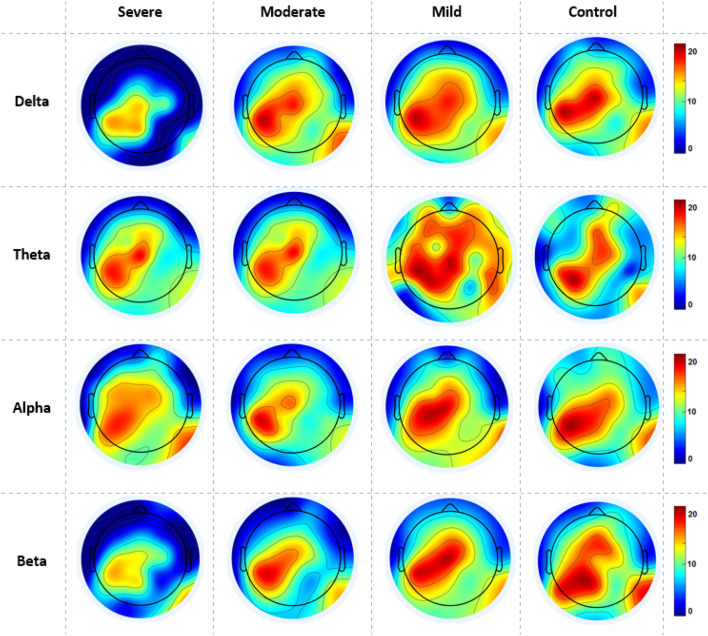
A mean nodal strength in resting-state for all four SAD groups (severe, moderate, mild, and HCs) and frequencies (delta, theta, alpha, and beta). Red color indicate higher nodal strength; White color indicate lower nodal strength. The topographic maps were created with EEGLAB function (the venerable topoplot). This includes a 2D grid representation of the scalp and nodal strength values as color-coded dots or contours at the corresponding scalp locations (10-20 system).

Figure 6 gives the average cortical NS values across all electrodes of the SAD groups and HCs at the RSN in all frequency bands. SAD groups and HCs have shown significant differences in theta, alpha, and beta bands with (*p* > 0.02, *p* > 0.01, and *p* > 0.05), respectively. Severe and moderate SAD have shown enhanced NS of connectivity at the PCC cortex compared to mild and HC groups in theta band. In addition, it has been found increased NS at the left mPFC for severe and moderate groups in the alpha band and increased NS in the right mPFC in beta band. Furthermore, mild and HC groups showed aberrant cortical activity at the precuneus and LPC in low-frequency bands (delta and theta) compared with high SAD severities. The increment of nodal strength of connectivity reflects higher information transfer between the cortical nodes. The mean and standard deviation along with statistical findings of nodal strength are reported in Table 3.

**Table 3:** Graph theory measures compassion for different frequency ranges in HC and SAD groups (ANOVA test, *p* < 0.05).

**Graph theory measure**	**Group/statistical analysis**	**Delta**	**Theta**	**Alpha**	**Beta**
Nodal degree	Severe	0.434 ± 0.047	0.299 ± 0.088	0.39 ± 0.06	0.334 ± 0.047
	Moderate	0.283 ± 0.063	0.54 ± 1.95	0.303 ± 0.07	0.1893 ± 0.063
	Mild	0.248 ± 0.062	0.345 ± 1.95	0.179 ± 0.06	0.143 ± 0.072
	Control	0.162 ± 0.053	0.03 ± 0.036	0.071 ± 0.05	0.261 ± 0.062
	*F*	1.762	1.378	1.013	1.491
	*P*-value	0.227	0.0253*	0.0501*	0.298
Nodal strength	Severe	6.105 ± 0.885	4.627 ± 1.678	6.132 ± 1.133	6.338 ± 0.933
	Moderate	4.873 ± 1.152	6.273 ± 1.721	5.30 ± 1.325	5.491 ± 1.210
	Mild	4.461 ± 1.182	5.073 ± 1.721	3.65 ± 1.416	3.994 ± 1.335
	Control	3.264 ± 1.128	4.901 ± 3.965	1.695 ± 1.086	2.557 ± 1.144
	*F*	1.461	3.904	4.07	1.173
	*P*-value	0.17	0.02*	0.01*	0.05*
Clustering coefficient	Severe	0.102 ± 0.008	0.315 ± 0.010	0.188 ± 0.009	0.009 ± 0.001
	Moderate	0.091 ± 0.007	0.231 ± 0.049	0.100 ± 0.007	0.007 ± 0.001
	Mild	0.086 ± 0.008	0.181 ± 0.049	0.084 ± 0.015	0.0107 ± 0.001
	Control	0.070 ± 0.011	0.116 ± 0.012	0.054 ± 0.029	0.0158 ± 0.002
	*F*	0.08	2.025	1.802	0.2339
	*P*-value	0.862	0.0114*	0.014*	0.751
Local efficiency	Severe	0.105 ± 0.008	0.144 ± 0.033	0.113 ± 0.001	0.108 ± 0.009
	Moderate	0.107 ± 0.008	0.132 ± 0.011	0.113 ± 0.001	0.112 ± 0.008
	Mild	0.104 ± 0.010	0.132 ± 0.011	0.102 ± 0.01	0.102 ± 0.014
	Control	0.088 ± 0.018	0.126 ± 0.011	0.064 ± 0.03	0.071 ± 0.021
	*F*	1.213	4.535	5.923	1.100
	*P*-value	0.219	0.05*	0.04*	0.387
Modularity	Severe	2.100 ± 0.711	2.667 ± 0.958	1.466 ± 0.507	1.53 ± 0.51
	Moderate	2.301 ± 1.235	2.233 ± 0.935	2.066 ± 0.739	1.53 ± 0.51
	Mild	1.831 ± 0.833	2.233 ± 0.935	1.766 ± 0.085	2.07 ± 01.08
	Control	2.667 ± 1.124	1.500 ± 0.509	2.33 ± 1.093	1.93 ± 0.86
	*F*	3.234	9.606	6.151	3.721
	*P*-value	0.013*	0.001*	0.01*	0.013*

The asterisk indicates a statistical significance between the means.

#### 3.4.3. Measure of segregation

**Figure 7:**
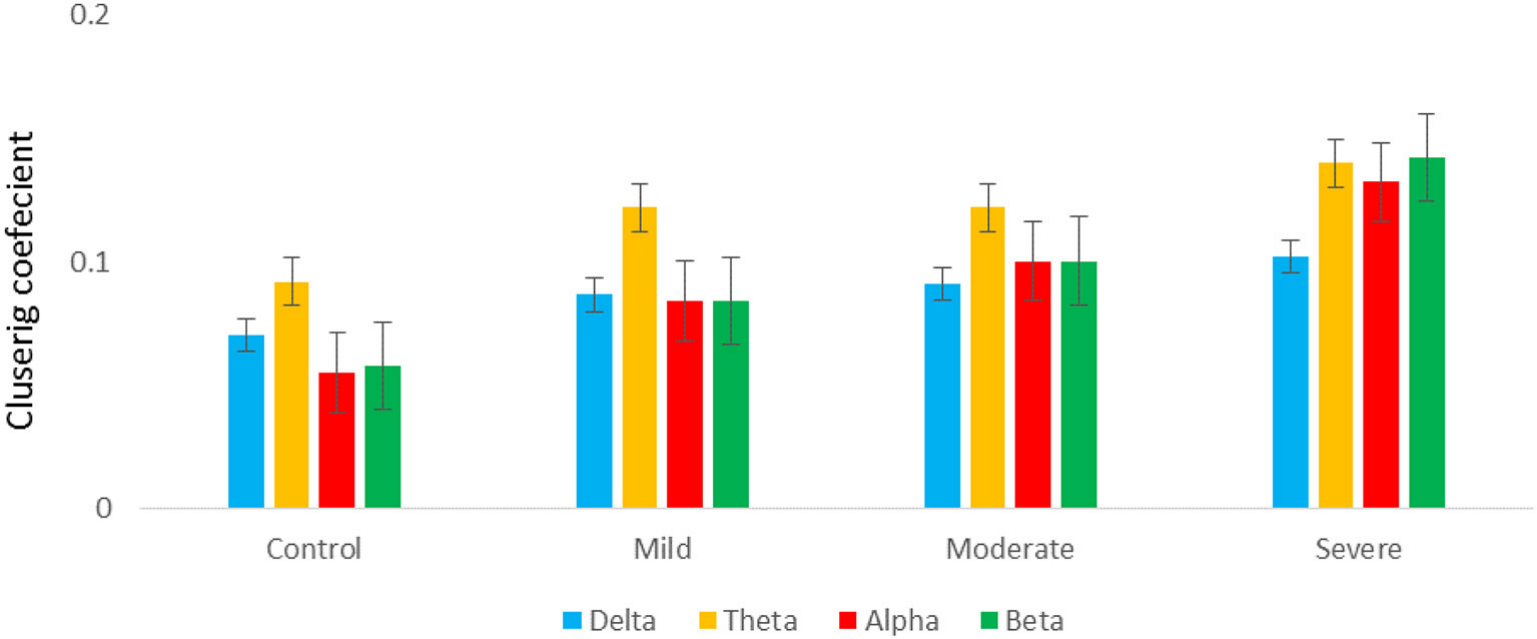
A graph's clustering coefficient values for all SAD groups for 4 frequency.

**Figure 8:**
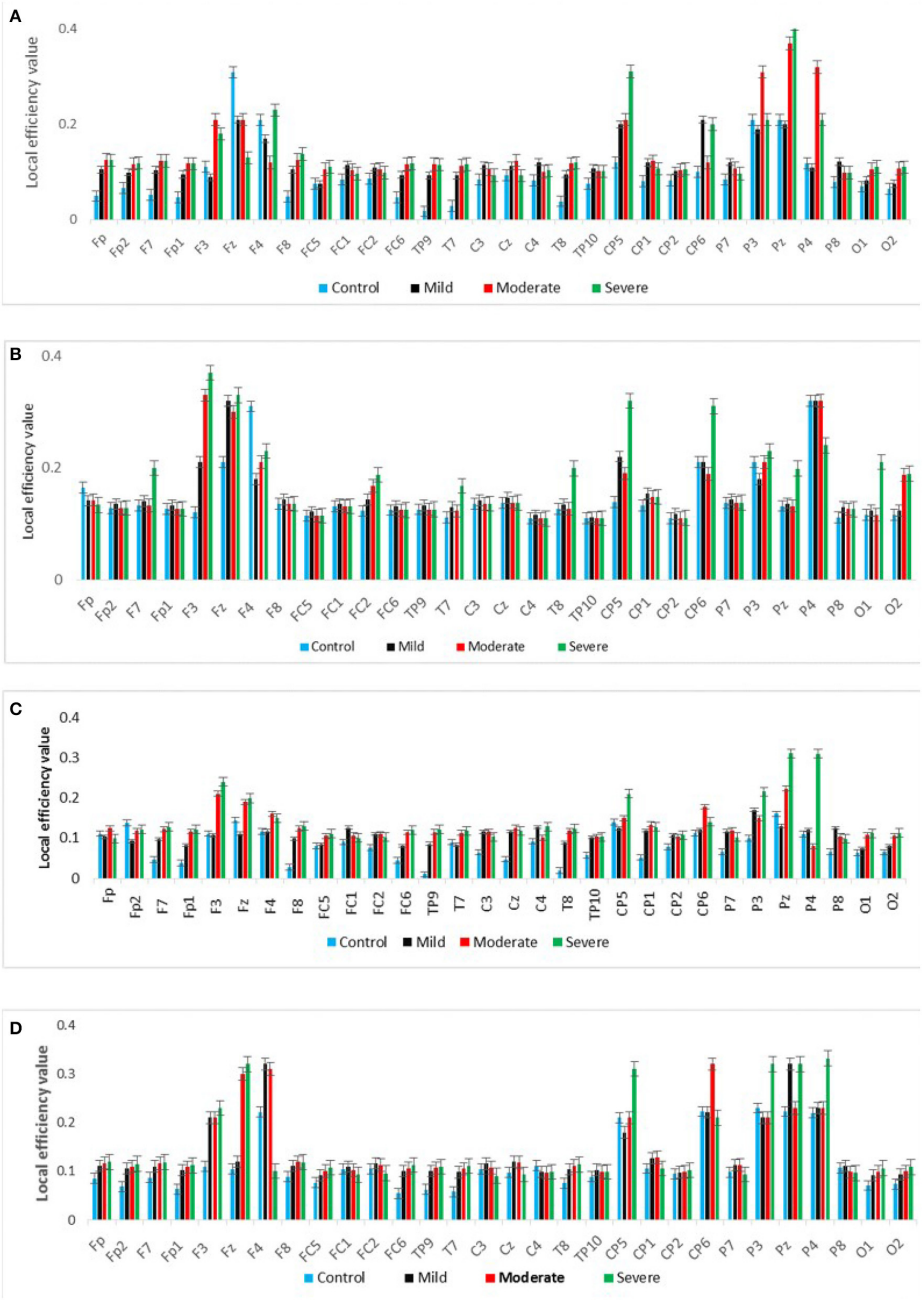
A graph's local efficiency for all SAD groups for four frequency ranges [delta **(A)**, theta **(B)**, alpha **(C)**, and beta **(D)**].

Figure 7 shows the averaged CC of EC at each node in the graph between SAD groups in four different frequency bands in RSN. In theta and alpha bands, the CC values show a significant difference between SAD groups (*p* > 0.011 and *p* > 0.014), and the mean of CC was found to be greater in severe and moderate. A significant CC indicates the efficiency of the network at the local scale. Conceivably, the graph analysis of CC using PDC connectivity values evidenced more significant nodes. Additionally, Figure 8 shows the averaged local efficiency (LE) values of connectivity for three SAD groups and HC in different frequency bands. Significant differences were found between SAD groups and HCs in theta and alpha (*p* > 0.011 and *p* > 0.014), respectively. Similarly, no statistically significant differences are found for LE in all delta and beta bands. The averaged LE across all 30 cortical nodes was decreased in HC and mild groups. The nodes were subjected to mPFC (Fz), PCC (Pz), right posterior transverse temporal (T8), and LPC (P4). In high-frequency bands (alpha and beta), local efficiency is increased for all SAD groups compared to low-frequency bands (delta and theta).

#### 3.4.4. Modularity

**Figure 9:**
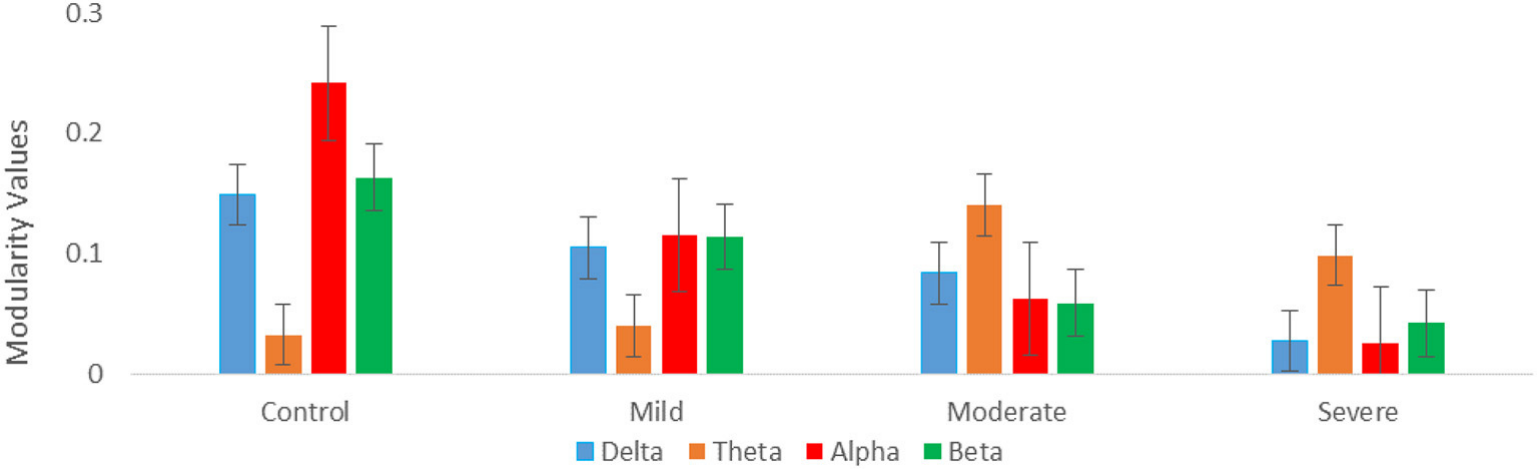
Graph theory measures; mean modularity values for all SAD groups for four frequency ranges (delta, theta, alpha, and beta).

Regarding optimal community structure Modularity (*Ci*) of theta connectivity, a significant difference was found between all SAD groups with (*p* > 0.013), (*p* > 0.013), (*p* > 0.001, 0.01), and (*p* > 0.013) in delta, theta, alpha, and beta, respectively. Table 3 exhibits the mean values and standard deviations for modularity measures in SAD and HC groups. Furthermore, another measure of modularity (Q) has been found to be increased HC group in the alpha band. Together, severe and moderate groups have shown the highest Q values in theta band as shown in Figure 9.

### 3.5. Correlations between connectivity and symptom severity

**Figure 10:**
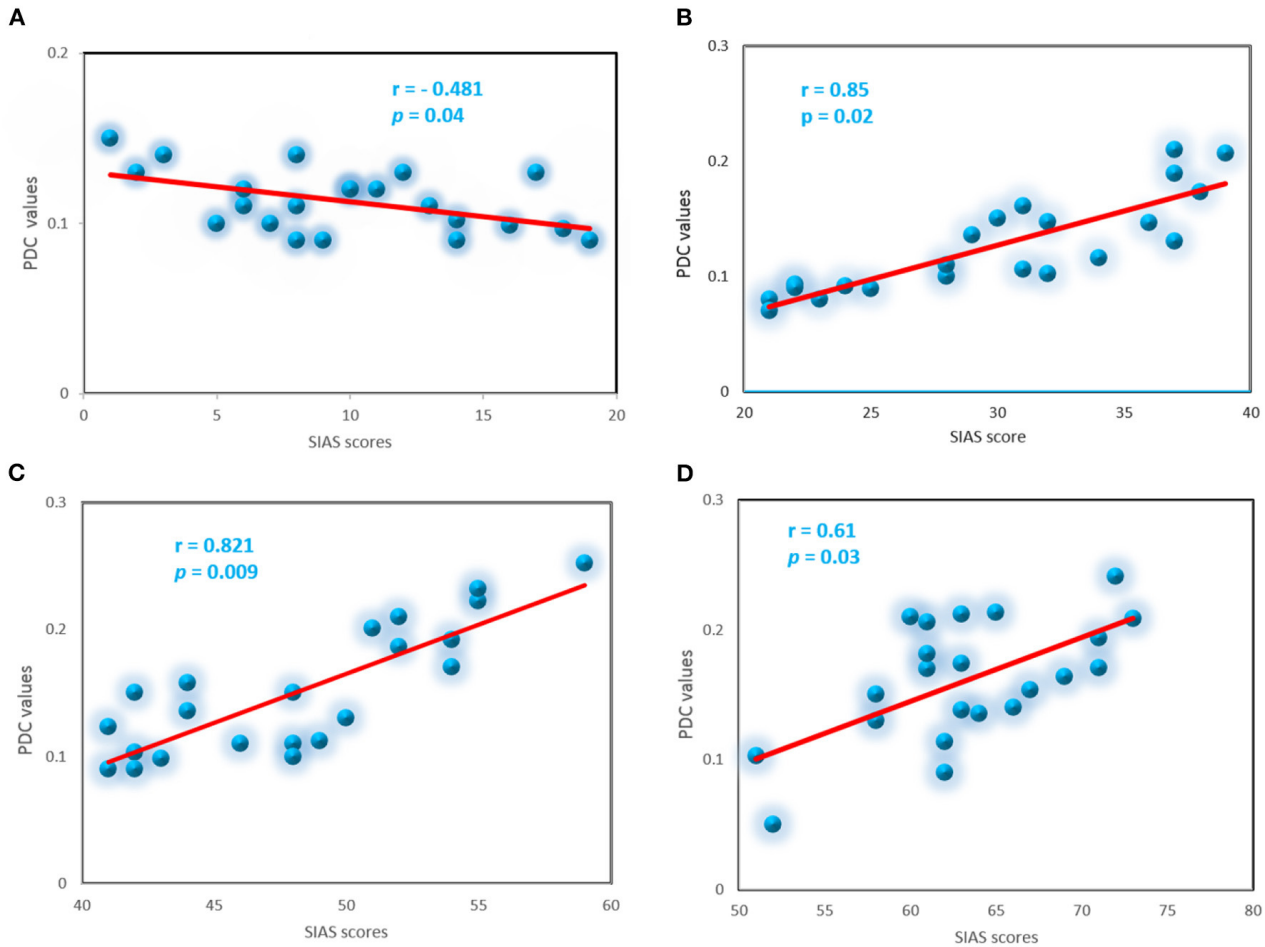
Correlation between the PDC and severity symptoms SIAS scores for the SAD groups in alpha band, control **(A)**, mild **(B)**, moderate **(C)**, and severe **(D)**.

To explore whether abnormal effective connectivity in SAD correlated with SAD symptom severity, a Pearson correlation test was computed between PDC values and SIAS scores. Relationship between self-assessment and PDC values analysis here is restricted to the alpha-band due to its observed effects and sensitivity in human brain dysfunctions. Within the SAD groups, the PDC values were positively correlated with SAD symptom severity measures with (*r* = 0.85, *p* < 0.02), (*r* = 0.82, *p* < 0.009), and (*r* = 0.61, *p* < 0.03) in mild, moderate, and severe groups, respectively. HC group exhibited negative correlation between the PDC values and self-report assessment of SAD (*r* = −0.481, *p* < 0.04). PDC values did not correlate with SIAS anxiety level in the other frequency bands. Figure 10 exhibited the correlation between PDC values and symptom severity in alpha band.

### 3.6. Classification

In this study, inputs constructed by the two main features (PDC and graph theory features) are both examined, by feeding them to different machine learning algorithms. In particular, we first classified the severity of SAD using PDC values with five different machine learning algorithms. The features extracted by PDC algorithm revealed maximum accuracy in classifying three classes of SAD from HC class with SVM classifier in theta frequency band (accuracy 83.66%, sensitivity 94.27%, and specificity 93.23%), and (accuracy 82.79%, sensitivity 93.25%, and specificity 92.12%) in alpha band.

Furthermore, we have applied a graphical theory network measure to construct feature vectors for machine learning classification. The features extracted from the combination of PDC and graph theories are found to outperform the PDC features alone in classification performance. Graphical theory classification using SVM classifier exhibited maximum accuracy in alpha band (accuracy 92.78%, sensitivity 93.25%, and specificity 92.12%) and (accuracy 91.66%, sensitivity 99.27%, and specificity 97.23%) in theta band. The training accuracy and validation accuracy are found to show robust and similar performance in the applied models, which indicates model's flexibility (fit model).

The overall classification findings in terms of the average accuracies, sensitivities, specificities, and standard deviations of the proposed algorithms are presented in Table 4 for the PDC algorithm and Table 5 for PDC + graphical networks. The results suggest that PDC and graph measures are competent to obtain intrinsic and distinctive features from EEG data to better diagnose SAD severity and significantly improve classification performance.

**Table 4:** Comparisons of the average accuracies and standard deviations (%) of EEG-based SAD severity classification by using PDC values.

**Features**	**Classifiers**	**Evaluation metrics**	**Delta**	**Theta**	**Alpha**	**Beta**
PDC features	KNN	Sensitivity	93.52 ± 0.61	98.31 ± 0.52	98.85 ± 0.83	91.32 ± 2.23
		Specificity	92.82 ± 1.77	95.91 ± 4.01	94.32 ± 0.79	87.82 ± 0.79
		Accuracy	75.00 ± 4.40	70.83 ± 2.67	76.54 ± 2.76	74.37 ± 3.58
	LDA	Sensitivity	94.83 ± 1.75	95.66 ± 1.044	99.32 ± 1.38	96.83 ± 0.75
		Specificity	93.00 ± 4.44	93.62 ± 3.56	97.87 ± 3.46	94.61 ± 2.49
		Accuracy	67.00 ± 7.71	62.83 ± 7.26	68.08 ± 6.59	66.58 ± 7.39
	NB	Sensitivity	97.18 ± 1.87	97.12 ± 1.0	85.71 ± 16.07	92.15 ± 2.32
		Specificity	96.28 ± 2.17	96.33 ± 3.01	85.18 ± 1.51	94.23 ± 0.77
		Accuracy	79.70 ± 2.52	71.47 ± 2.07	79.75 ± 1.91	87.10 ± 2.11
	DT	Sensitivity	92.71 ± 7.10	89.30 ± 9.23	93.91 ± 11.12	91.80 ± 0.86
		Specificity	91.28 ± 6.20	81.84 ± 8.09	91.14 ± 4.51	92.82 ± 5.31
		Accuracy	75.95 ± 4.15	72.12 ± 3.68	75.62 ± 2.24	75.00 ± 2.74
	SVM	Sensitivity	91.27 ± 6.20	94.27 ± 6.13	93.25 ± 4.27	91.53 ± 6.47
		Specificity	90.13 ± 6.81	93.23 ± 6.81	92.12 ± 5.91	90.19 ± 9.28
		Accuracy	79.29 ± 5.93	83.66 ± 6.72	82.79 ± 7.86	80.08 ± 10.94

**Table 5:** Comparisons of the average accuracies and standard deviations (%) of EEG-based SAD severity classification by using PDC and graph theory values.

**Features**	**Classifiers**	**Evaluation metrics**	**Delta**	**Theta**	**Alpha**	**Beta**
GT+PDC features	KNN	Sensitivity	99.52 ± 0.61	98.31 ± 0.52	92.85 ± 0.83	95.30 ± 2.13
		Specificity	97.82 ± 1.77	95.91 ± 4.01	94.32 ± 0.79	99.82 ± 0.79
		Accuracy	82.00 ± 4.40	87.83 ± 2.67	88.54 ± 2.76	84.37 ± 3.58
	LDA	Sensitivity	94.83 ± 1.75	99.66 ± 1.044	99.32 ± 1.38	99.83 ± 0.75
		Specificity	93.00 ± 4.44	95.62 ± 3.56	97.87 ± 3.46	98.61 ± 2.49
		Accuracy	77.00 ± 7.71	77.83 ± 7.26	78.08 ± 6.59	66.58 ± 7.39
	NB	Sensitivity	97.18 ± 1.87	97.12 ± 1.0	85.71 ± 16.07	96.98 ± 2.10
		Specificity	96.28 ± 2.17	96.33 ± 3.01	85.18 ± 1.51	99.23 ± 0.77
		Accuracy	82.70 ± 2.52	82.47 ± 2.07	83.75 ± 1.91	83.10 ± 2.11
	DT	Sensitivity	92.71 ± 7.10	89.30 ± 9.23	93.91 ± 11.12	99.80 ± 0.86
		Specificity	91.28 ± 6.20	81.84 ± 8.09	91.14 ± 4.51	96.34 ± 2.03
		Accuracy	85.95 ± 4.15	89.12 ± 3.68	89.62 ± 2.24	86.00 ± 2.74
	SVM	Sensitivity	91.27 ± 6.20	99.27 ± 0.13	95.25 ± 4.27	91.53 ± 6.47
		Specificity	90.13 ± 6.81	97.23 ± 1.81	94.12 ± 5.91	90.19 ± 9.28
		Accuracy	83.29 ± 5.93	91.66 ± 6.72	92.78 ± 7.86	86.08 ± 10.94

## 4. Discussion

To the best of our knowledge, this is the first study that used EEG-based whole-brain effective connectivity and graph theory analysis along with machine learning models to segregate the severity of SAD. The main objective of the present study was to verify whether the intrinsic connectivity EEG and graphical theory network measures of RSNs can be used to categorize the severity level of SAD (severe, moderate, mild, and HC) and evaluate the presence of a relation between the neural alterations in brain and disease severity. We hypothesized that PDC and graph theory analysis could also elucidate the neurobiological mechanisms underlying pathophysiology in SAD. Noticeably, the EEG data analysis and the demonstrated qualitative and quantitative results are generalized to young participants only who aged between 18 and 25 years old.

In alpha, the predominant oscillation at rest (54), there was a significantly enhanced directed connection in the averaged PDC values in individuals with SAD (severe and moderate groups) more than HCs. The increased information flow was found mainly in occipital and left partial cortices. In addition to alpha, theta frequency bands exhibited significant differences among the three groups of SAD and HC. An enhanced information flow and increased connections were found in the mPFC, medial-frontal cortex, left lateral parietal cortex, and PCC compared with HC group. However, no group effects were revealed in beta or delta frequencies. Group differences in alpha and theta bands were observed in the absolute power, PDC and graph theory measures. Severe SAD group has shown the highest values of PDC compared to HCs subjects who exhibited reduced cortical information flow in different cortical regions. Evidence of alterations in RSN alpha regional power has been reported in SAD studies (55, 56), and abnormal alpha power or functional connectivity has been found in other mental disorders like depression, Alzheimer's disease, and suicidal behaviors (57–59). Consequently, our findings are in line with the above-mentioned studies of alpha rhythm involvement in internalizing conditions. Consequently, functional theta connectivity was found to be deviant in SAD compared to HC individuals (23). Conceivably, compared to HC and mild groups, high severe SAD groups have shown enhanced effective theta connectivity in mPFC, midline, LPC, and occipital cortices. Commonly, theta rhythm in RSN, produced by cortical brain neurons located in the mPFC cortex (consists of Brodmann areas 24, 32, and 33) is related to mental task performance and cognitive attentiveness reflecting concentrated referential processing in HC individuals (60). Genuinely, the higher connectivity at higher levels of SAD is believed to reflect greater monitoring of physiological reactions to threats. Higher connectivity in HCs can be associated with lower levels of severity symptoms and may reflect more efficient information processing and better communication between brain regions involved in emotion regulation, cognitive control, and social cognition. In individuals with SAD, certain brain regions may be overactive or underactive, leading to disruptions in communication and coordination between these regions. Improvements in brain connectivity may indicate a normalization of these patterns, which can in turn lead to reduced symptoms of SAD (61).

We suggest that SAD patients are involved in excessive memory processing. Theta oscillation and functional connectivity changes in temporal lobe disorder with comorbid depression has been previously reported (62). We found a rise in information flow from the dorsolateral prefrontal cortex to the posterior cortex. In the context of mental brain disorders, theta functional connectivity in the dorsal frontoparietal connection is thought to indicate goal-directed attention (63). It is noteworthy that most of the neural changes we found in our analysis were located within the default mode network (DMN). Our research identified a set of eight operationally active cortical regions, which included the precuneus/posterior cingulate cortex (PCC), anterior cingulate cortex (ACC), medial prefrontal cortex (mPFC), and lateral parietal inferior gyri (LPC) (64). The DMN shows heightened brain connectivity during rest or when engaging in referential processing, compared to goal-directed tasks.

Graph theory network measures mainly comprise predominate local large-scale connectivity reflected by directed weighted measures of high ND, NS, CC, LE, and modularity. All the measures of these features represent information processing efficacy at a low cost. Here, we found a significant result in theta and alpha frequency bands, but not in delta and beta bands. SAD patients have shown greater graph theory measures in the mPFC, LPC, ACC, and occipital cortex than HCs. The greater values in SAD patients of graph theory measures suggest stronger interactions between the executive networks (active brain regions) and higher-order association areas. These results confirm that the higher level of anxiety strongly influences the causal information flow between brain regions. Therefore, we believe that graph theory measures in the whole-brain network can be considered as effective biomarkers for SAD recognition. Table 6 presents a comparison and superiority of our proposed model over the other recent SAD works. It presents the type of applied classifiers, number of classes, type graph theory measures, and the accuracy performance.

**Table 6:** Comparison of the proposed method in terms of accuracy (ACC) with recent machine learning and graph theory measures.

**References**	**Year**	**Feature**	**Subjects**	**Classes**	**Channels**	**Graph theory**	**Classifier**	**ACC**
Xing et al. (23)	2017	Weighted Lag Index	64	2	34	CC, CPL	SVM	–
Zhu et al. (65)	2017	Functional connectivity	84	2	fMRI	CC, PL	SVM	71.4
Zhang et al. (66)	2015	Functional connectivity	80	2	fMRI	Cortical hubs	SVM	76.25
Chen et al. (67)	2021	Spectral Power	34	3	8	–	SVM	87.18
Alaei et al. (68)	2023	Directed Transfer Function	24	2	19	centrality Strength	SVM	91.66
Our proposed method	2021	Effective connectivity	88	4	32	ND,NS LE, CC T and M	SVM	92.78

The accuracy is reported in %.

The higher severe SAD groups (severe and moderate), compared with the HC group demonstrated greater CC, modularity, and LE than HC and mild groups. Furthermore, ND and NS were found to be higher in severe and moderate groups relative to HC and mild groups in all frequency bands. Accordingly, PDC and graph theory network measures in theta band (4–7 Hz) and alpha band (8–12 Hz) are suggested to be potential biomarkers for SAD (23). Moreover, in three groups of SAD groups, but not the HC group, there was a significant positive correlation between alpha PDC values and symptom severity (SIAS reports). HC group exhibited a significant negative correlation between PDC in the alpha band and symptom severity. In addition, no significant correlation was found in the other frequency bands (delta, theta, and beta). Previously, multiple studies that used PDC in RSN have reported abnormal neural changes in internalizing psychopathologies (69).

In addition, the features extracted from the information flow between brain regions alone and features from PDC with their corresponding graph theory measures successfully classified three SAD classes and HC class with high accuracy. PDC features alone showed classification accuracy with 79.29, 83.66, 82.79, and 80.08% in delta, theta, alpha, and beta bands, respectively, between severe, moderate, mild, and HC classes using SVM classifier. However, the combined features (PDC + graph theory measures) achieved higher accuracy performance with 83.29, 91.66, 92.78, and 86.08% in delta, theta, alpha, and beta bands, respectively, using SVM classifier. The results showed that the SVM outperformed other classifiers in all frequency bands, as seen in Table 5. The improvement in accuracy by combining PDC and graph theory matrices is consistent with previous research that utilized directional information flow with functional connectivity in neurodevelopmental analysis, resulting in remarkable accuracy prediction (47, 70).

There are some important limitations of this study that must be mentioned. First, several patients with comorbid conditions were involved in this analysis. Whilst SAD was their central identification, comorbid mental disorders such as major depressive disorder and generalized SAD may have impacted the results. Therefore, new analyses of SAD individuals with and without comorbid mental disorders are needed. Second, our analysis is restricted to the young population with different sample size between females and males,thus future studies may be specified to the analysis of different populations in different settings with balanced sample size. Third, our analysis was based on whole-brain connectivity, thus, alternate network analysis may be considered for future work (e.g., DMN network, dorsal attention network (DAN), and salience network) to draw the specific functions of specific regions in the brain to assess the severity of SAD. Fourth, our study was restricted to resting-state only, future studies can be dedicated to estimating the severity of SAD in different states.

## 5. Conclusion

In the present work, an essential contribution is the integrated use of the PDC and graph theoretical measures to estimate the severity of SAD and compare them with HCs. The whole-brain results exhibited altered RSN information flow between brain regions in SAD groups. These alterations in causal information flow in the RSN connectivity may be associated with the detected severity symptoms in SAD patients. Moreover, impairments in neural circuits involving mPFC and, PCC, and LPC may have a role in SAD psychopathology. As mentioned above, the combination of PDC and graph theory techniques enhances the possibility of detecting relevant features of the effective brain networks. To this end, we have achieved a high classification accuracy in distinguishing three classes of SAD and HC by using machine learning based on a combination of information flow features and graph theory measures. Taken together, our results suggest RSN brain connectivity may serve as a neurophysiological biomarker for SAD diagnosis.

## Data availability statement

The original contributions presented in the study are included in the article/supplementary material, further inquiries can be directed to the corresponding authors.

## Ethics statement

The studies involving human participants were reviewed and approved by Universiti Kuala Lumpur Royal College of Medicine Perak (UniKL RCMP). The patients/participants provided their written informed consent to participate in this study.

## Author contributions

NK and AA-E: study conception and design. AA-E: data collection. AA-E and AAA-S: analysis and interpretation of results. AA-E and AA-S: draft manuscript preparation. NY, FA-S, and MA-H: experimental design and psychological revisions. All authors reviewed the results and approved the final version of the manuscript.
