# Corrigendum: Machine learning for the detection of social anxiety disorder using effective connectivity and graph theory measures

**DOI:** 10.3389/fpsyt.2023.1257713

**Published:** 2023-07-24

**Authors:** Abdulhakim Al-Ezzi, Nidal Kamel, Amal A. Al-Shargabi, Fares Al-Shargie, Alaa Al-Shargabi, Norashikin Yahya, Mohammed Isam Al-Hiyali

**Affiliations:** ^1^Centre for Intelligent Signal & Imaging Research (CISIR), Electrical and Electronic Engineering Department, Universiti Teknologi PETRONAS, Bandar Seri Iskandar, Perak, Malaysia; ^2^College of Engineering and Computer Science, VinUniversity, Hanoi, Vietnam; ^3^Department of Information Technology, College of Computer, Qassim University, Buraydah, Saudi Arabia; ^4^Faculty of Engineering, Abu Dhabi University, Abu Dhabi, United Arab Emirates; ^5^Department of Information Technology, Universiti Teknlogi Malaysia, Skudai, Malaysia

**Keywords:** EEG, graph theory analysis, social anxiety disorders, machine learning, effective connectivity, partial directed coherence, support vector machine, event related potential

In the published article, there was an error in the Funding statement. The correct Funding and Acknowledgment statements appear below.

